# Cluster of imported cutaneous leishmaniasis due to *Leishmania major* among travellers returning from central Tunisia, France, October 2025 to May 2026

**DOI:** 10.2807/1560-7917.ES.2026.31.32.2600605

**Published:** 2026-08-13

**Authors:** Morgane Andreoletti, Emilie Guemas, Eléna Charpentier, Judith Fillaux, Alexis Valentin, Pamela Chauvin, Antoine Berry, Xavier Iriart

**Affiliations:** 1 Université de Toulouse, CHU Toulouse, Hôpital Purpan, Service de Parasitologie-Mycologie, Toulouse, France; 2 Université de Toulouse, CHU Toulouse, Institut Toulousain des Maladies Infectieuses et Inflammatoires (Infinity), CNRS UMR5051, INSERM UMR1291, Hôpital Purpan, Service de Parasitologie-Mycologie, Toulouse, France

**Keywords:** leishmaniasis, *Leishmania major*, Tunisia, outbreak, PCR, diagnosis

## Abstract

Between October 2025 and May 2026, 28 patients with cutaneous leishmaniasis caused by *Leishmania major* were identified at Toulouse University Hospital, France, contrasting with fewer than three cases diagnosed annually in 2019–2024. Epidemiological investigations linked 24 cases to recent travel to Tunisia, with 19 reporting exposure in the governorate of Sidi Bouzid, endemic for zoonotic cutaneous leishmaniasis. These findings suggest increased transmission in central Tunisia and reinforce the need for surveillance of imported leishmaniasis in Europe.

Cutaneous leishmaniasis (CL), caused by *Leishmania* parasites and transmitted by sand flies, remains a major public health concern, with an estimated 600,000 to 1 million [1] new cases occurring annually worldwide. The Mediterranean basin constitutes one of the main endemic regions, and Tunisia reports among the highest incidences in northern Africa [2]. In contrast, CL is uncommon in metropolitan France, where most cases are imported. Here, we describe a cluster of imported *Leishmania major* infections diagnosed in Toulouse, south-western France, following travel to Tunisia during summer/early autumn 2025.

## Event detection

In Toulouse, we observed an increase in detection of *Leishmania* species from cutaneous specimens tested with a *Leishmania* genus-specific real-time PCR assay [3]. Between 2025 and 2026, 35 of 114 (30.7%) skin specimens were positive for *Leishmania* spp. Species identification, done by sequencing [3], revealed *L. major* in 28 specimens: 17 were diagnosed in 2025 and 11 between January and May 2026. This represented more than a sixfold increase in patients with *L. major* compared with the previous 6 years.

## Retrospective investigation

We reviewed all samples submitted to the Toulouse University Hospital parasitology laboratory for the investigation of cutaneous leishmaniasis between 1 January 2019 and 31 May 2026. Cutaneous specimens consisted of skin biopsies and lesion swabs. Samples tested for the diagnosis of visceral leishmaniasis (blood and bone marrow) were excluded.

**Figure 1:**
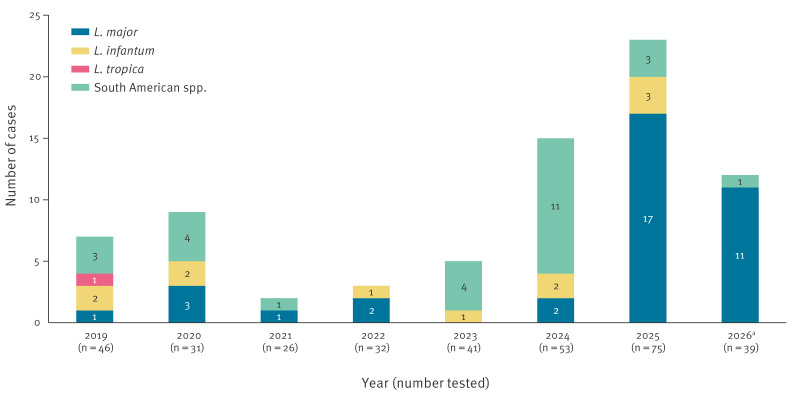
Annual number of cutaneous leishmaniasis diagnoses by PCR, Toulouse University Hospital, France, 2019–2026 (n = 343)

^a^ January–May 2026.

Between January 2019 and May 2026, 76 of 343 (22.2%) diagnostic PCR tests for suspected cutaneous leishmaniasis were positive for *Leishmania* spp. Between 2019 and 2024, the annual number of patients with laboratory-confirmed cutaneous leishmaniasis remained relatively stable, ranging from 2 to a peak of 15 in 2024 with the latter owed to a marked increase of imported cases from South America (Figure 1). Overall, 41 of 229 (17.9%) PCR tests performed during this period were positive; *L. major* was identified in only nine patients with no more than three annually.

The number of tests performed for cutaneous leishmaniasis is indicated in parentheses below each year.

Isolates with South American *Leishmania* species (n = 27): *L. guyanensis* (n = 19), *L. peruviana* (n = 3), *L. lainsoni* (n = 2), *L. amazonensis* (n = 1), *L. mexicana* (n = 1) and *L. panamensis* (n = 1).

## Epidemiological investigation

The unexpected increase in patients diagnosed with cutaneous leishmaniasis prompted an epidemiological investigation. A standardised questionnaire was administered to collect information on travel history, probable place of exposure, travel dates, purpose of travel, clinical presentation and treatment.

Among the 28 patients with confirmed *L. major* infection diagnosed between October 2025 and May 2026, 24 reported recent travel to Tunisia and three to Morocco, while one patient had no documented travel history. Detailed information on the travel destination within Tunisia was available for 19 travellers, all of whom had stayed in the Sidi Bouzid Governorate landlocked in central Tunisia and endemic for *L. major* [4]. Information on the purpose of travel was available for 15 patients, 14 of whom reported travelling regularly to Sidi Bouzid, primarily to visit friends and relatives.

**Figure 2:**
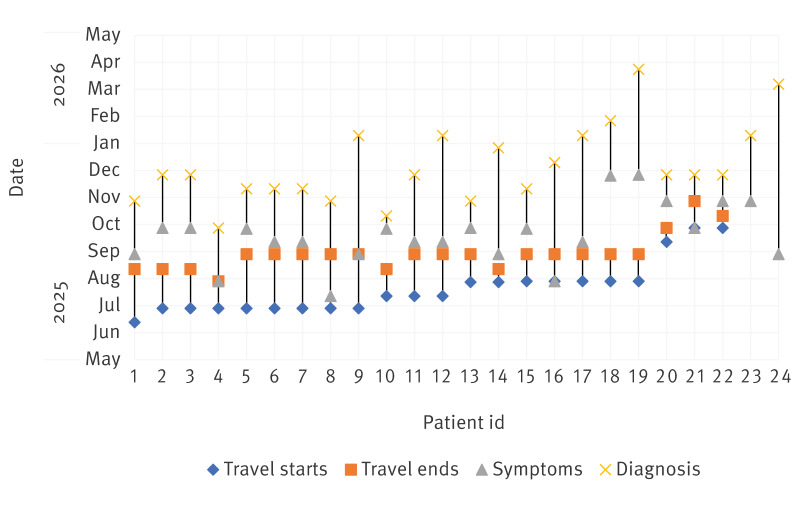
Timeline of travel to Tunisia, symptom onset and laboratory diagnosis of patients with cutaneous leishmaniasis, 2025–2026 (n = 24)

Travel dates to Tunisia varied from late June to late October 2025, with 19 travellers staying in Tunisia in July–August (Figure 2). Symptom onset ranged from the time of travel to several weeks after return. The interval between presumed exposure and laboratory confirmation varied from 31 to 213 days (median: 87.5 days; IQR: 65–132 days). This variability is consistent with the incubation period of zoonotic cutaneous leishmaniasis and reflects the often delayed recognition of the disease in non-endemic settings.

Fifteen patients were males and nine were females. The median age was 14 years (mean: 26 years; range: 1.5–77 years), reflecting the predominance of paediatric cases. None of the patients reported direct contact with rodents, the principal reservoir of *L. major*. However, all described staying in rural environments, away from urban centres, and seven recalled insect bites during their stay, supporting exposure to infected sand flies in rural transmission settings.

## Clinical picture

Among the 24 patients, the number of lesions ranged from 1 to 15 (median: 3). Six patients presented with a single lesion, whereas eight had five or more lesions. Lesions predominantly involved exposed body sites, mainly the upper and lower limbs (20/24) and the face (10/24), including the cheeks, nose, forehead, eyelids and periorbital region. Less frequently affected sites were the trunk (5/24), buttocks (2/24), neck (2/24,) and scalp (1/24).

Notably, six patients aged < 13 years (age range: 4–12.5 years) had multiple lesions (range: 3–15; median: 6) on both the limbs and in the face. This observation is consistent with previous reports indicating that in zoonotic CL caused by *L. major*, multiple lesions are common [5] and facial involvement is more frequent in children [6].

The most common clinical manifestations were ulcerative or ulcerocrusted lesions (19/24), frequently surrounded by an inflammatory raised border and occasionally associated with erythematous plaques. Some patients had erythematous papules and hyperkeratotic or ulcerated nodules. Three patients had a complicated disease: two with a secondary bacterial infection, one associated with cellulitis of the leg and the other with marked inflammatory facial oedema, and one patient had regional lymphadenopathy associated with facial swelling.

## Clinical management

Clinical management was performed according to current French recommendations, considering the number of lesions, size, localisation and species identification [7,8].

Seventeen patients received topical paromomycin. Four patients required systemic treatment with liposomal amphotericin B because of multiple lesions, lesion size or cosmetically sensitive site. One patient received fluconazole after an immediate hypersensitivity reaction to amphotericin B. Two patients were lost to follow-up before treatment initiation. No severe treatment-related complications were reported.

## Discussion

We describe an approximately sixfold increase in *L. major* diagnoses in Toulouse over a period of 8 months compared with previous years. During this period, 24 patients returning from travel to Tunisia in 2025 were diagnosed with confirmed *L. major* infection. This observation is particularly noteworthy in the national context, as only 207 cases of imported cutaneous leishmaniasis were reported in France in 2024, including 41 among patients returning from the Maghreb region [9].

The observed cluster of imported cutaneous leishmaniasis suggests a probable increase in *L. major* transmission in central Tunisia during 2025. Alternative explanations, such as changes in travel patterns, activities or healthcare-seeking behaviour among travellers returning from Tunisia, should also be considered. We identified no major changes in referral patterns, diagnostic practices or molecular species identification at our centre during the period.

The epidemiological characteristics observed are consistent with zoonotic CL caused by *L. major* which is endemic in central and southern Tunisia [4]. In 2022, the Sidi Bouzid Governorate reported an incidence of 669.7 cases per 100,000 inhabitants [4]. Transmission involves phlebotomine sand flies and rodent reservoirs. Environmental factors, such as waste accumulation, urban expansion and climatic conditions, may favour both vector and reservoir populations, thereby increasing transmission intensity [10].

The temporal distribution of cases suggests exposure during summer/early autumn 2025 followed by diagnosis several months later, in line with the known incubation period of CL. Most patients travelled regularly to the Sidi Bouzid Governorate to visit friends and relatives, reflecting the strong demographic links between France and Tunisia. Tunisians represent the fourth largest immigrant community in France (approximately 340,000 first-generation immigrants in 2023) [11]. The predominance of paediatric patients is consistent with this travel profile, as children and adolescents commonly accompany their families when visiting friends and relatives in endemic areas. Although CL is rarely life-threatening, its public health impact should not be underestimated. Lesions may persist for months, lead to permanent scarring and cause substantial psychosocial burden. Furthermore, management of complex cases may require systemic treatments associated with significant toxicity and healthcare costs.

Our findings also demonstrate the value of routine molecular surveillance. Species identification allowed rapid recognition of an unusual increase in imported *L. major* infections that would not have been detected through generic leishmaniasis reporting alone. Enhanced surveillance of imported CL and timely species identification may contribute to the early detection of epidemiological changes occurring in endemic regions and improve awareness among healthcare professionals in Europe.

## Conclusion

Clinicians in France and other European countries should consider CL in patients presenting with chronic ulcerative or crusted skin lesions after travel to North Africa. Healthcare professionals providing pre-travel advice should reinforce preventive messages for travellers to endemic countries, particularly individuals visiting friends and relatives in endemic areas. Travellers should be informed of the risk of cutaneous leishmaniasis and advised to prevent sand fly bites using insect repellents, insecticide-treated bed nets and protective clothing during evening and nighttime exposure. Continued surveillance of imported cases will be important to determine whether the increased transmission observed in 2025 persists during the current travel season.

## Data Availability

All data generated or analysed during this study are included in this article.
